# Enhanced antibacterial activity of roxithromycin loaded pegylated poly lactide-co-glycolide nanoparticles

**DOI:** 10.1186/2008-2231-20-92

**Published:** 2012-12-12

**Authors:** Mona Noori Koopaei, Mohamad Shahab Maghazei, Seyed Hossein Mostafavi, Hossein Jamalifar, Nasrin Samadi, Mohsen Amini, Soheyl Jafari Malek, Behrad Darvishi, Fatemeh Atyabi, Rassoul Dinarvand

**Affiliations:** 1 Novel Drug Delivery Lab, Faculty of Pharmacy, Tehran University of Medical Sciences, Tehran 1417614411, Iran; 2 Nanotechnology Research Centre, Faculty of Pharmacy, Tehran University of Medical Sciences, Tehran, Iran; 3 Department of Drug and Food Control, Faculty of Pharmacy, Tehran University of Medical Sciences, Tehran, Iran; 4 Department of Medicinal Chemistry, Faculty of Pharmacy, Tehran University of Medical Sciences, Tehran, Iran

**Keywords:** Roxithromycin, PLGA, Pegylation, Nanoparticles, Antibacterial

## Abstract

**Background and the purpose of the study:**

The purpose of this study was to prepare pegylated poly lactide-co-glycolide (PEG-PLGA) nanoparticles (NPs) loaded with roxithromycin (RXN) with appropriate physicochemical properties and antibacterial activity. Roxithromycin, a semi-synthetic derivative of erythromycin, is more stable than erythromycin under acidic conditions and exhibits improved clinical effects.

**Methods:**

RXN was loaded in pegylated PLGA NPs in different drug;polymer ratios by solvent evaporation technique and characterized for their size and size distribution, surface charge, surface morphology, drug loading, in vitro drug release profile, and in vitro antibacterial effects on *S. aureus, B. subtilis, and S. epidermidis*.

**Results and conclusion:**

NPs were spherical with a relatively mono-dispersed size distribution. The particle size of nanoparticles ranged from 150 to 200 nm. NPs with entrapment efficiency of up to 80.0±6.5% and drug loading of up to 13.0±1.0% were prepared. In vitro release study showed an early burst release of about 50.03±0.99% at 6.5 h and then a slow and steady release of RXN was observed after the burst release. In vitro antibacterial effects determined that the minimal inhibitory concentration (MIC) of RXN loaded PEG-PLGA NPs were 9 times lower on *S. aureus*, 4.5 times lower on *B. subtilis*, and 4.5 times lower on *S. epidermidis* compared to RXN solution. In conclusion it was shown that polymeric NPs enhanced the antibacterial efficacy of RXN substantially.

## Introduction

Infectious diseases were the important reason of death worldwide at the beginning of the 20th century [1]. An introduction of antibacterial agents leads to the decreases in mortality and morbidity from infectious diseases over the last century. These days, though, resistance to antibiotics has been reaching a critical level, invalidating major antibacterial drugs that are currently used in the clinic [2,3]. Exploring antibacterial NPs is one of the new efforts in addressing this challenge, to which microbial pathogens may not be able to develop resistance. For instance, in recent studies it has been suggested that some metal nanostructures are recognized to have antibacterial activities, which is utilized in controlling infectious diseases [4].

The advantages of NP-based antibacterial drug delivery include improved solubility of poorly water-soluble drugs, sustained and stimuli-responsive drug release, which eventually lowers administration frequency and dose, and prolonged drug half-life and systemic circulation time. Moreover, minimized systemic side effects via targeted delivery of antibacterial drugs as well as combined, synergistic, and resistance-overcoming effects via co delivery of multiple antibacterial drugs can be achieved using NP carriers [5,6].

Among different nanoparticulate systems, polymeric NPs have several unique characteristics for antibacterial drug delivery. Firstly, particle properties such as size, zeta potentials, and drug release profiles can be precisely tuned by selecting different polymer lengths, surfactants, and organic solvents during the preparation process. Secondly, polymeric NPs are structurally stable and can be prepared with a narrow size distribution. Thirdly, the surface of polymeric NPs can be functionalized by groups that can be chemically customized with either drug moieties or targeting ligands [5].

Among the different polymers developed to formulate polymeric NPs, PLGA has attracted considerable attention due to its attractive properties: (i) biodegradability and biocompatibility, (ii) FDA and European Medicine Agency approval in drug delivery systems for parenteral administration, (iii) well described formulations and methods of production adapted to various types of drugs e.g. hydrophilic or hydrophobic small molecules or macromolecules, (iv) protection of drug from degradation, (v) possibility of sustained release, (vi) possibility to modify surface properties to provide stealth properties and/or better interaction with biological materials and (vii) possibility to target NPs to specific organs or cells [7].

Poly ethylene glycol (PEG) has been frequently used for the surface treatment of polymeric NPs to create a stealth layer that prolongs the circulation lifetime of NPs in the blood stream. Specifically, the PEG coating forms a hydration layer that retards the reticuloendothelial system (RES) recognitions of NPs through sterically inhibiting hydrophobic and electrostatic interactions with plasma proteins [8]. This minimizes the interface with phagocytic cells and increasing the blood circulation time. Another attractive characteristic of PEG, with admiration to its use for mucosal drug administration, is associated to its mucoadhesion promoting result, a mechanism of chain penetration across the polymer mucosa interface may be the reason for this phenomenon.[9].

Roxithromycin (RXN), a semi-synthetic 14-memberedring macrolide antibiotic derived from erythromycin, has a molecular weight of 837.06. It possesses improved pharmacokinetic properties such as high serum concentrations and a long half-life of about 13 h. Furthermore, this it is more resistant to gastric acid hydrolysis. Because of these properties, together with its spectrum of activity, RXN appears to offer advantages for the treatment of respiratory tract infections [10] . RXN has bitter taste and is very slightly soluble in aqueous medium. The oral bioavailability of RXN is 50% and the low bioavailability of the drug is mainly due to poor aqueous solubility and dissolution behavior [11]. Ciprofloxacin loaded PLGA NPs showed increased antibacterial effect [12]. In another study, it was reported that rifampicin loaded PLGA NPs are remarkably more effective against gram-positive bacteria [13].

In previous study, RXN microspheres with polymers were prepared successfully by the emulsion solvent diffusion method to cover its bitter taste [14]. Preparation and characterization of complexes of RXN with â-cyclodextrin (BCD), hydroxypropyl â-cyclodextrin (HPBCD) to increase the solubility, improve the dissolution properties and improve the antibacterial activity has been also reported so as to increase the efficacy of this narrow spectrum antibiotic [11].

In this study RXN loaded PEG-PLGA NPs were prepared to improve its antibacterial effect. Their physicochemical characters, drug release and in vitro antibacterial effects against *S. aureus*, *B. subtilis*, and *S. epidermidis* were determined.

## Methods

### Materials

RXN powder (USP) was kindly donated by Shifa PharMed Co, Iran. PLGA (lactide:glycolide, 50:50; Resomer RG 504H, molecular weight 48,000) was purchased from Boehringer Ingelheim (Ingelheim, Germany). Polyvinyl alcohol (molecular weight 22,000) and bifunctional NH2-PEG-OH (average molecular weight 10000) were purchased from Sigma-Aldrich (St Louis, MO). 1-Ethyl-3-(3-dimethylaminopropyl)carbodiimide (EDC), N-hydroxysuccinimide (NHS), Poly vinyl alcohol (PVA), with molecular weight of 95,000 from Acros Organics (Geel, Belgium) and acetone from Merck (Darmstadt, Germany) were used. Mueller-Hinton broth and Mueller-Hinton agar (both from Merck) media were used for microbiological tests. *S.aureus* (ATCC : 6538) , *B. subtilis* (ATCC : 6633), and *S. epidermidis* (ATCC : 12228) were obtained from the stock culture of Department of Drug and Food Control, Faculty of Pharmacy, Tehran University of Medical Sciences, Iran. In all experiments reverse osmosis (RO) purified water was freshly prepared prior to use. All other materials used were of analytical or HPLC grade.

### Synthesis of PEG-PLGA

Hydroxylate functionalized PEG-PLGA was synthesized by conjugation of OH-PEG-NH2 to carboxylic group of PLGA [15]. NHS (135 mg, 1.1 mmol) in the presence of EDC (230 mg, 1.2 mmol) was added to a stirred solution of PLGA-COOH (5 g, 0.14 mmol) in methylene chloride (10 mL). The mixture was stirred for 3 hours at 25°C. PLGA-NHS was precipitated with ethyl ether (5 mL) and repeatedly washed in an ice-cold mixture of ethyl ether and methanol to remove the residual NHS. After drying under vacuum, PLGA-NHS (1 g, 0.059 mmol) was dissolved in chloroform (4 mL) followed by addition of NH2-PEG-OH (250 mg, 0.074 mmol) and N,N-diisopropylethylamine (28 mg, 0.22 mmol). The copolymer was precipitated with cold methanol after 12 hours and washed with the same solvent (35 mL) to remove unreacted PEG [15]. The resulting PEG-PLGA block copolymer was dried under vacuum and used for NP preparation without further treatment. The following are the main nuclear magnetic resonance (NMR) peaks of the sample:

1H-NMR (CDCl3 at 300 Hz) d 5.2 (m, (OCH(CH3)C(O)OCH2C(O)n-(CH2CH2O)m), 4.8 (m, (OCH(CH3)C(O)OCH2C(O)n-(CH2CH2O)m), 3.7 (s, (OCH(CH3)C(O)OCH2C(O)n-(CH2CH2O)m), 1.6 (d, (OCH(CH3)C(O)OCH2C(O))n-(CH2CH2O)m.

### Preparation of roxithromycin-loaded pegylated PLGA nanoparticles

PEG-PLGA NPs loaded with RXN with different ratios of the drug were prepared by a solvent evaporation method [16,17]. Briefly, known amounts of polymer and RXN were added into a mixture of 1 ml DCM:acetone (3:10), which was suitably stirred to ensure that all material was dissolved. This organic phase was emulsified with 6.7 ml of aqueous solution of PVP (0.5% w/v) by sonication using a probe sonicator (Misonix S4000-010, USA) at amplitude 10 for 5 min. NPs were formed immediately, and gently stirred at room temperature for 5 h to evaporate the organic solvent. The resulting NP suspension was then ultracentrifuged (15,000 rpm for 20 min at room temperature) using a Sigma 3K30 centrifuge. After centrifugation, the NP precipitate was washed using the same volume of distilled water as the supernatant, and again centrifuged at 15,000 rpm for 15 minutes. The washing process was repeated three times in order to remove the adsorbed drugs. To Control of NP during post-formulation treatment, the size and drug loading of NP were studied through the storage in solid-state three months after freeze-drying.

### Particle size and zeta potential measurement

The particle size distribution and poly dispersity index (PDI) of NPs were determined by Photon Correlation Spectroscopy (PCS) using a Zetasizer nanoZS (Malvern Instruments, Worcestershire, UK) at 25°C. Zeta potential of NPs was also measured by the same instrument. Before the measurement, all samples were diluted ten times with deionized water to prevent inter particle aggregation. All the measurements were triplicate and Mean±SD is reported.

### Drug loading and entrapment efficiency

Lyophilized NPs (2.5 mg) were dissolved in 1 mL of acetonitrile and shaken lightly followed by sonication for 6 minutes. Thereafter, 2 mL of methanol was added to precipitate the polymer. The sample was filtered and the drug quantity in the filtrate was determined by high-pressure liquid chromatography. Drug loading was determined as the ratio of drug content of NPs to the total weight of the NPs. The entrapment efficacy was obtained as the ratio of the amount of RXN incorporated in NPs to the amount used for the preparation of NPs [18]. The experiments were carried out in triplicate.

### In vitro drug release

The RXN release profile at pH 7.4 in phosphate-buffered saline and at pH 5.2 in acetate buffer was studied. Five mg of freeze-dried RXN loaded NPs was poured into screw-capped tubes and suspended in 10 mL of phosphate-buffered saline pH 7.4 or acetate buffer pH 5.2. The tubes were placed in a water bath maintained at 37 ± 0.5°C and shaken at 90 cycles/min. At fixed time intervals, the tubes were taken from the water bath and centrifuged at 21,000 g for 15 min. The NPs were resuspended in 10 mL of fresh buffer and placed back into the water bath to continue the release measurements. An aliquot of 9 mL was taken from the supernatant. A volume of 1 mL of methanol was added to precipitate any remaining PLGA, centrifuged for 15 min at 13255 rpm, and analyzed by high-pressure liquid chromatography. The experiments were carried out in triplicate [13].

HPLC analysis was performed at 37°C, using a Knauer apparatus (model K-1001, WellChrom, Berlin, Germany) equipped with a reversed-phase C18 column (25 cm × 0.46 cm internal diameter, pore size 5 μm; Teknokroma, Barcelona, Spain) and eluted isocratically with Mix 307 volumes of acetonitrile and 693 volumes of a 49.1 g/l solution of ammonium dihydrogen phosphate adjusted to PH 5.3 with dilute sodium hydroxide solution. The flow rate was fixed at 1.5 ml/min and detection was obtained by UV detection at 205 nm. The linear regression coefficient determined in the range 0.05–10 μg/ml was 0.9994 (n = 6). The method sensitivity was 0.05 μg/ml (signal to noise ratio, 3:1) [19].

### Differential scanning calorimetry (DSC)

DSC was performed to investigate the physical status of RXN in the NPs. DSC scans of RXN, PEG-PLGA, physical mixture of PEG-PLGA and RXN and RXN-loaded NPs were performed on a Mettler DSC 823e equipped with Mettler STARe system software for the data acquisition. The samples were scanned at a speed of 5°C/min in a 30–250°C temperature range.

### Surface morphology

Scanning electron microscopy (SEM, Philips XL 30, Philips, The Netherlands) was used to determine the shape and surface morphology of the NPs produced. NPs were coated with gold under vacuum before scanning electron microscopy.

### Antibacterial efficacy test

Broth microdilution technique was utilized to determine the MIC of RXN loaded NPs. RXN solution with the same concentration of NPs and drug free NPs, prepared exactly similar to RXN NPs except no drug was added against *S. aureus*, *B. subtilis*, and *S.epidermidis* were used as positive and negative control groups. Drug free NPs were used to ensure the carrier and the ingredients do not have any antibacterial activity. The bacterium obtained in lyophilized form which was cultured in Luria Bertuni agar medium (Scharlau, Spain) after suspending in sterile distilled water. The plate incubated for 24 h at 37°C. Single colony from the plate was transferred into 4 ml fluid of LB medium and incubated over night at 37°C and 200 rpm in shaking incubator. The cells were harvested by centrifugation at 3000 rpm (Behdad, Iran) for 15 min at 4°C. Subsequently, they were washed twice and resuspended in Ringer solution to provide the concentration range of 105–106 CFU/ml for broth dilution method (National Committee for Clinical Laboratory Standards, 1996). A quantity of 12 tubes for each sample and each microbial strain were employed respectively. In each tube 1ml of medium was placed and was autoclaved at 121°C for 15 min. After sterilization, 1 ml of sample was added to the first tube and mixed [13].

### Statistical analysis

One-way analyses of variance (ANOVA) were performed for comparison of the results. A value of P < 0.05 was considered to be significant.

## Results and discussion

### Synthesis of PEG-PLGA

**Figure 1:**
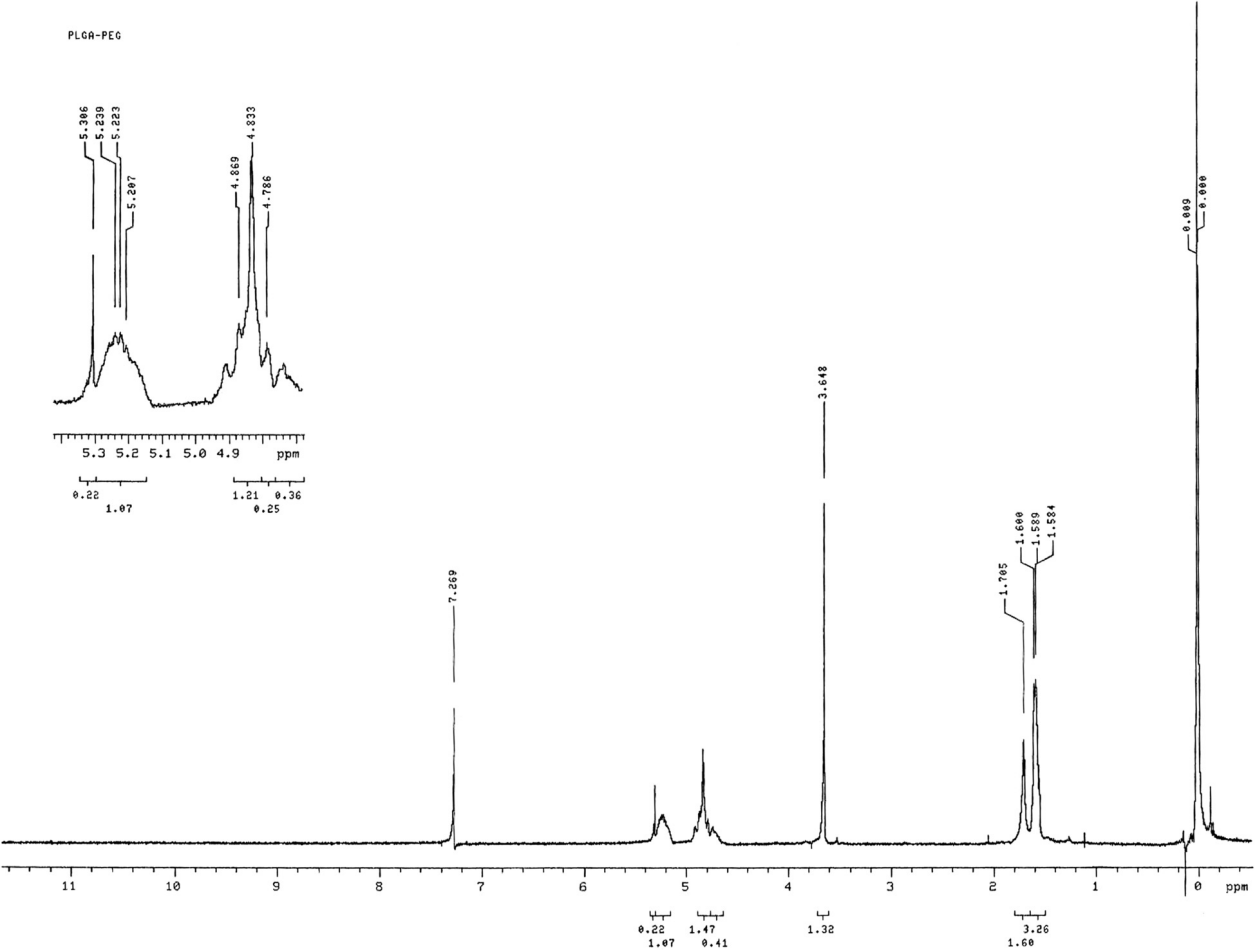
^**1**^**H NMR spectrum of PEG–PLGA copolymer.**

In this study PEG-PLGA was synthesized and characterized. The basic chemical structure of PEG-PLGA copolymer was confirmed by 1H-NMR (Figure 1). This figure is very similar to the previously reported spectrum and confirms the synthesis of the PLGA-PEG copolymer. One of the prominent features is a peak at 3.7 ppm, matching the methylene groups of PEG. Overlapping doublets at 1.6 ppm are attributed to the methyl groups of the D- and L-lactic acid repeat units. The multiples at 5.2 ppm and 4.8 ppm correspond to the lactic acid -CH and the glycolic acid -CH, respectively, with the high complexity of the peaks resulting from different D-lactic, L-lactic glycolic acid sequences in the polymer backbone [15].

### Preparation and characterization of roxithromycin-loaded nanoparticles

**Figure 2:**
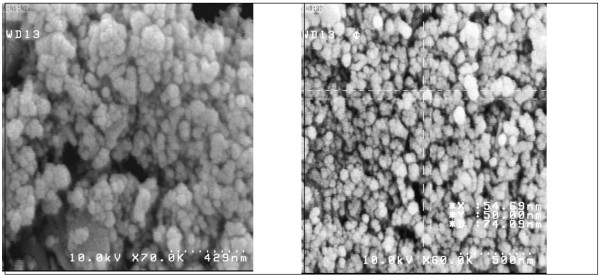
SEM micrographs of nanoparticles showing the shape and the surface characteristics of NPs.

Nanoparticles were prepared by sonication-solvent evaporation method. Entrapment efficiencies, drug loading, size, morphology and zeta potential of RXN pegylated NPs are reported in the Table 1. As shown in Table 1, the size of NPs was in the range of 150–200 nm. . Increasing the amount of internal phase, containing drug and polymer, increased the size of nanoparticles. This may be the result of enhanced viscous forces resisting droplet break down by sonication. The viscous forces is in opposition to the shear stresses in the organic phase and the final finishing size and size distribution of particles depends on the net shear stress available presented for droplet breakdown. Narrow range of size distribution was observed for NPs and the prepared NPs exhibited a relative monodisperse distribution. As seen in Figure 2, SEM micrograph of the RXN loaded NPs confirms that NPs are in spherical shape with smooth surfaces (Figure 2). All formulations appeared to be homogenous irrespective of their compositions. The particle size is an important parameter, as the biopharmaceutical properties of nanoparticles can be influenced by its physicochemical properties. The pegylation of nanoparticles increases the blood circulation of nanoparticles which suitable for IV administration [19,20]. The negative zeta potential of the PLGA nanoparticles tends to limit their capability to interact with negatively charged plasmids and intracellular uptake. Pegylation of PLGA helps to adjust the surface charge of the nanoparticles to become less negative. In this study, PLGA-PEG NPs showed less negative zeta potential (-1.9±0.11) than PLGA NPs (-25.4±0.24). It has been proved that the amount of phagocytosis is the lowest when zeta potential is closer to zero [21].

**Table 1:** Physicochemical characteristics of the roxithromycin nanoparticles

**Sample**	**RXN-PLGA (mg)**	**Mean diameter ± SD (nm)**	**PDI ± SD**	**Zeta potential ± SD (mV)**	**Drug loading (%)**	**Entrapment efficiency (%)**
**S1**	4 -25	152±8.4	0.09±0.10	-1.88±0.72	8.8±1.3	64.0±4.2
**S2**	5 -25	178±6.3	0.13±0.03	-2.63±0.53	10.5±1.1	73.0±3.8
**S3**	8.2- 25	196±10.5	0.11±0.05	-6.23±2.10	13.0±1.0	80.0±6.5
**S4**	10 -20	177±6.7	0.09±0.05	-2.48±0.36	11.0±2.1	34.0±5.5
**S5**	10- 10	181±11.6	0.93±0.12	-4.02±1.64	12.5±1.8	41.0±4.3

The table also shows an increase in the amount of PLGA resulted in an increase in the drug entrapment. This may be attributed to the higher viscosity of the internal organic phase for higher PLGA ratios, which in turn would decrease the diffusion coefficient of the drug.

**Figure 3:**
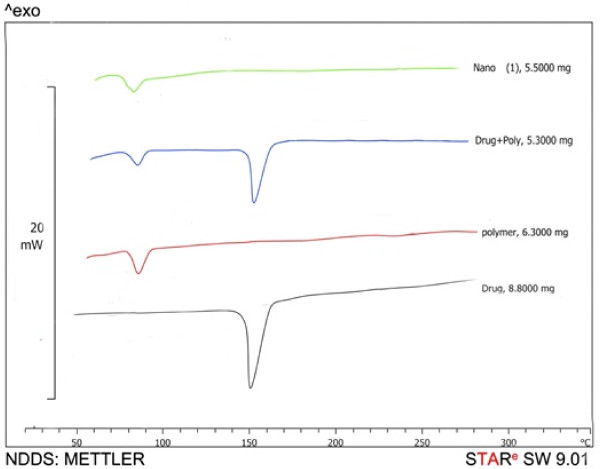
DSC thermograms of roxithromycin, PEG-PLGA, physical mixture of roxithromycin and polymer, and roxithromycin loaded PEG-PLGA nanoparticles.

DSC was carried out to investigate the effect of the process on thermal behavior of formulations [20]. The DSC scans of samples and the melting point data are presented in Figure 3. At the mentioned scanning rate (5°C/min) pure RXN powder had an endothermic peak at 125°C. However, no melting peak could be detected for the NP formulations. This shows that DTX in the NPs is in an amorphous or disordered crystalline phase, miscible in polymeric NPs. The reduction in enthalpy of the physical mixture of drug–polymer is due to the presence of smaller amount of drug in the mixture in comparison with pure drug.

NPs when freeze dried with sucrose (10% w/w), as cryoprotectant, were physically stable. No significant changes in size, zeta potential or drug content was detected after freeze dried NPs kept at 4°C for three months.

### In vitro roxithromycin release

**Figure 4:**
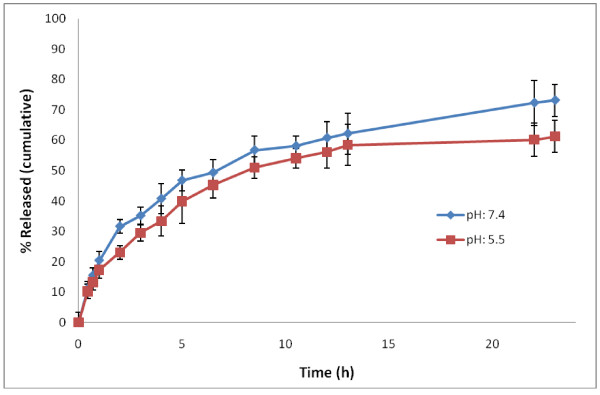
**In vitro roxithromycin release profiles from nanoparticles in acetate buffer (pH 5.5, 37°C) and phosphate-buffered solution (pH = 7.4, 37°C). **Data are represented as mean ± standard deviation (n = 3).

The profile of in vitro release of RXN from NPs in the acetate buffer (pH 5.2) and phosphate-buffered saline (pH 7.4) within first day is depicted in Figure 4. During the first hours of release, an early burst release greater than 50% was detected. A slow and steady release of RXN was observed after the burst release. The burst release may be due to the release of RXN loaded on the surface or just beneath the surface of the NPs. Then the sustained release is mainly due to the diffusion of drug molecules through the polymeric matrix of the NPs.

### Antibacterial activity of the nanoparticle suspensions

**Figure 5:**
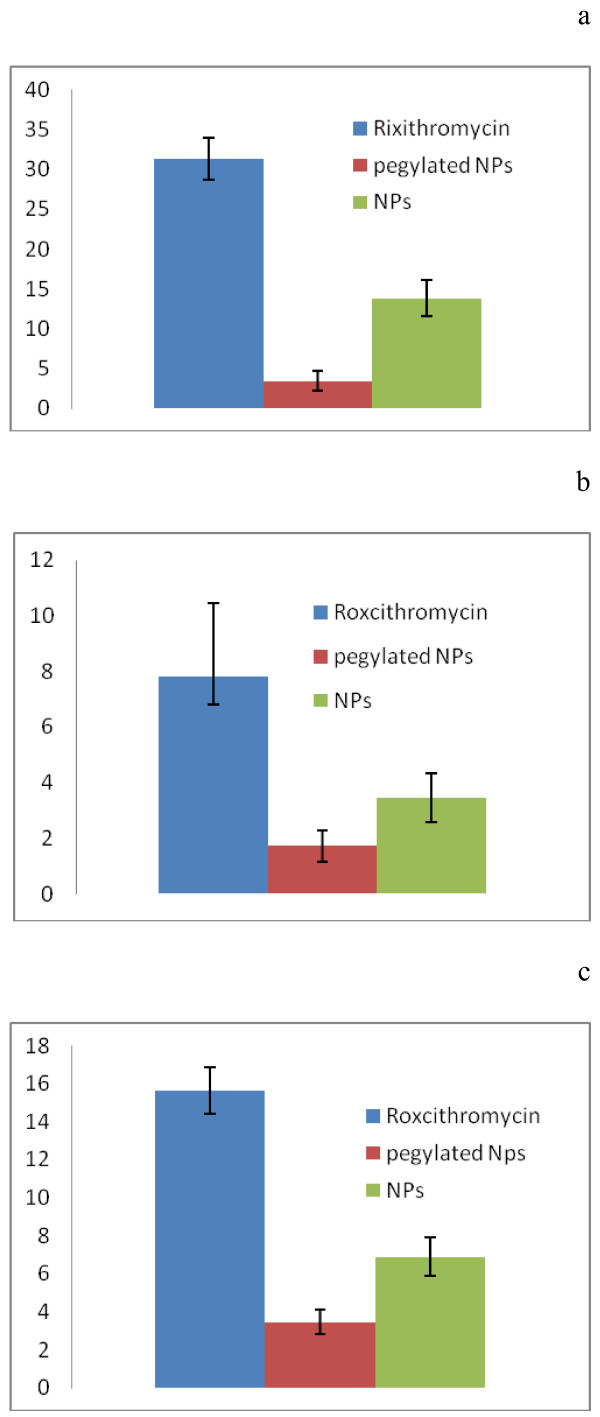
**Minimum inhibitory concentrations (MICs) (±SD) of the intact roxithromycin, roxithromycin loaded nanoparticle and pegylated NPs suspensions against *S. aureus *(a),* S. epodermidis *(b) and B. subtilis (c) (n = 3).**

The MIC of PEG-PLGA formulations and RXN powder on *S. aureus, B. subtilis, and S. epidermidis* are reported in Figure 5. Drug free polymeric nanoparticles showed no antibacterial activity which indicates that none of the ingredients have any antibacterial effect. The MIC of RXN loaded PLGA NPs were 2.3 times lower on *S. aureus*, 2.2 times lower on *B. subtilis,* and 2.4 times lower on *S. epidermidis* compared to RXN solution. Consequently, in vitro anti-microbial activity of RXN PLGA NPs is better than the commercialized drug formulations. Additionally, The MIC of RXN loaded PEG-PLGA NPs were 9 times lower on *S. aureus*, 4.5 times lower on both *B. subtilis*, and *S. epidermidis* compared to RXN solution. The higher antibacterial effect of RXN PLGA NPs and PEG-PLGA NPs may have been resulted from higher bacterial adhesion of the NPs. In other studies enhanced antimicrobial activity from PLGA NPs containing antimicrobial agents has been reported [21,22]. The improved the therapeutic effect of the nanoparticulate antibacterial delivery systems may the result of increased drug concentration nearby its target. Moreover, improved penetration of NPs from biological membranes is also generally accepted. For RXN to be effective it should pass the bacterial membrane to reach its intra cellular site of action, subunit 50S of the bacterial ribosome to inhibits the transaction of peptides.

## Conclusion

In this study, RXN a semi-synthetic macrolide antibiotic was successfully loaded into PEG-PLGA NPs. MIC tests indicated that antibacterial activity of RXN NPs increased several times compared to RXN aqueous solution against *S. aureus*, *B. subtilis*, and *S. epidermidis*. This allows more efficient therapy compared with the antibiotic in its original form. However, more studies are necessary to be approved in animals to verify the pharmacological activities.

## Competing interest

The authors declare that they have no competing interests.

## Authors’ contributions

MNK conceived the study, synthesized the copolymer and drafted the manuscript. MSM carried out the experiments and assisted MNK in drafting the manuscript. SHM assisted in preparation of nanoparticles. NS Supervised the antibacterial studies. HJ participated in the antimicrobial studies. MA supervised the synthesis and characterization of copolymers. SJM and BD assisted in preparation and characterization of nanoparticles. FA supervised the preparation and characterization of nanoparticles. RD supervised the project and participated in its design and coordination and is the corresponding author of the manuscript. All authors read and approved the final manuscript.
